# A Population-Based Iterated Greedy Algorithm for Maximizing Sensor Network Lifetime

**DOI:** 10.3390/s22051804

**Published:** 2022-02-24

**Authors:** Salim Bouamama, Christian Blum, Pedro Pinacho-Davidson

**Affiliations:** 1 Mechatronics Laboratory (LMETR)—E1764200, Department of Computer Science, Ferhat Abbas University Sétif 1, Sétif 19000, Algeria; salim.bouamama@univ-setif.dz; 2 Artificial Intelligence Research Institute (IIIA-CSIC), Campus of the UAB, 08193 Bellaterra, Spain; 3 Department of Computer Science, Faculty of Engineering, Universidad de Concepción, Concepción 4070411, Chile; ppinacho@udec.cl

**Keywords:** population-based iterated greedy, disjoint dominating sets, lifetime maximization, wireless sensor networks

## Abstract

Finding dominating sets in graphs is very important in the context of numerous real-world applications, especially in the area of wireless sensor networks. This is because network lifetime in wireless sensor networks can be prolonged by assigning sensors to disjoint dominating node sets. The nodes of these sets are then used by a sleep–wake cycling mechanism in a sequential way; that is, at any moment in time, only the nodes from exactly one of these sets are switched on while the others are switched off. This paper presents a population-based iterated greedy algorithm for solving a weighted version of the maximum disjoint dominating sets problem for energy conservation purposes in wireless sensor networks. Our approach is compared to the ILP solver, CPLEX, which is an existing local search technique, and to our earlier greedy algorithm. This is performed through its application to 640 random graphs from the literature and to 300 newly generated random geometric graphs. The results show that our algorithm significantly outperforms the competitors.

## 1. Introduction

The field of wireless sensor networks (WSNs) has been enjoying a lot of attention in the last 20 years, both in research and in industry. This is certainly due to a multitude of different applications, including environmental monitoring, medical and health applications, security surveillance, transportation applications, structural applications, and emergency operations [1,2], just to name a few. WSNs are generally composed of a number of small devices equipped with one or more sensors, limited storage capacity, a limited power supply, and a radio communication system. As the weight of sensor devices often plays an important role, power supply—for example, by means of a battery—is generally limited, and battery-saving techniques are often used. The lifetime of a sensor device (in hours) may be computed by a division of the battery capacity (in Watt hours) and the average power drain (in Watts). However, the estimation of the lifetime of a sensor node is not a trivial task (see [3]) because energy consumption is the result of various factors, including, for example, the environmental temperature.

For these reasons, one of the principal research topics concerning WSNs is about network lifetime extension, while at the same time, providing sufficient communication reliability and sensing coverage. Note that in this context, the term *network lifetime* refers to the time during which the network is fully operational with respect to its tasks. In other words, the network lifetime is the time duration in which the overall sensing coverage is maintained. The lifetime of a WSN, therefore, heavily depends on the energy consumption of the individual sensor devices. Real-world examples of mechanisms for maximizing the network lifetime are manifold. They include, but are not limited to, smart agriculture monitoring [4], structural health monitoring [5], human activity monitoring [6], and road traffic monitoring [7]. Power-saving strategies such as the ones found in these examples can—according to [8]—be classified as belonging to one of the following groups:

- Sleep–wake cycling, also referred to as duty cycling. Here, sensor devices alternate between active and sleep mode;
- Power control through the adjustment of the transmission range of the radio communication systems;
- Routing and data gathering in an energy efficient way;
- Reduction of the amount of data transmissions and avoidance of useless activity.

In this paper, we will provide a technique for WSN lifetime extension that falls into the first category. More precisely, our technique makes use of the so-called communication graph. The nodes of this graph are the sensor devices belonging to the network (together with their locations). Two such nodes are connected by an edge if the corresponding sensor devices can communicate with each other via their radio communication systems. Note that sensor nodes have at least two tasks, also known as functionalities: (1) sensing data and (2) data processing and forwarding data to a base station. Between these two tasks, the latter one is by far more energy-intensive than the first one. In order to keep energy spending to a minimum, the nodes in a sensor network may be organized in terms of dominating sets in which the dominators (that is, the sensor nodes that form part of the dominating set) assume the task of cluster heads that take care of data processing and forwarding. However, sensor nodes are never switched off in this model. Those sensor nodes that do not form part of the (current) dominating set save energy by not having to perform data processing and forwarding. In contrast, data sensing is performed by all sensor nodes at all times. Such a model (or similar models) have been used in a wide range of papers in the literature; for example, see refs. [9,10,11]. For the above-mentioned reasons, our technique organizes the sensor nodes into a number of disjoint dominating sets, which are used—one after the other—for data processing and data forwarding.

### 1.1. Necessary Graph Theoretic Concepts

This paper makes use of some basic definitions and notations from graph theory. The most important ones are outlined in the following. (For a more profound introduction, the interested reader may refer to [12].) First, the communication graph is modeled by means of an undirected graph, G=(V,E), where *V* is the set of nodes, and *E* is the set of edges connecting (some of) the nodes. Hence, two nodes, v≠u∈V, which are connected by an edge, (v,u)∈E, are called *neighbors*. They may also be called *adjacent nodes*. The *open neighborhood* of a node, v∈V, is defined as N(v):={u∈V∣(v,u)∈E}. Sometimes, however, it is necessary to refer to the *closed neighborhood* N[v] of a node, v∈V, which is defined by adding *v* to N(v), that is, N[v]:=N(v)∪{v}. Next, the *degree* deg(v) of a node, v∈V, is defined as the cardinality of N(v), that is, deg(v):=|N(v)|. The concept of neighborhood can also be extended, from nodes to sets of nodes, in the following way. The open neighborhood, N(D), of a set, D⊆V, is defined as ${⋃}_{v\in D}N\left(v\right)$. Correspondingly, a node *v*’s closed neighborhood, N[D], is defined as N[D]:=N(D)∪D.

In this context, we also formally introduce the terms: dominating set, domatic partition, and domatic number of a graph. First, a subset, D⊆V, in which each node, v∈V\D, has at least one neighbor that forms part of *D* is called a *dominating set* of *G*. A node, v∈D—where *D* is a dominating set—is said to cover all its neighbors, in addition to itself. A trivial example of a dominating set of an undirected graph, G=(V,E), is *V*. Next, a set $D=\{D_{1},D_{2},\cdots ,D_{k}\}$ of subsets $D_{i}\subseteq V$ is called a *domatic partition* of a given, undirected graph, G=(V,E), if the following two conditions are fulfilled: (1) $D_{i}$ (i=1,…,k) is a dominating set of *G*, and (2) all sets of D are pairwise disjoint, that is, $D_{i}\cap D_{j}=\emptyset$ for all 1≤i<j≤k. If $D=\{D_{1},D_{2},\cdots ,D_{k}\}$ is a set of disjoint dominating sets of *G* with (1) ${⋃}_{D_{i}\in D}D_{i}\subset V$ and (2) $V\backslash {⋃}_{D_{i}\in D}D_{i}$ is not a dominating set, a domatic partition $D^{\prime }$ of *G* can easily be obtained by adding all vertices from $V\backslash {⋃}_{D_{i}\in D}D_{i}$, for example, to $D_{k}$. The *domatic number* of an undirected graph, G=(V,E), is defined as the size of the largest domatic partition of *G*, that is, the domatic number of *G* is $|D^{*}|$, where $D^{*}:=\mathrm{argmax}\left\{|D||D\;\mathrm{is}\;a\;\mathrm{domatic}\;\mathrm{partition}\;\mathrm{of}\;G\right\}$. It was shown in the literature that the domatic number of a graph, *G*, can be at most δ+1, where δ:=min{deg(v)∣v∈V}. The problem of identifying a domatic partition of an undirected graph, *G*, is sometimes called the *maximum disjoint dominating sets* (MDDS) problem.

### 1.2. Graph Problems Used to Model WSN Lifetime Maximization

The related literature offers different approaches for the maximization of the sensor network lifetime. Most of these approaches have modeled this problem either in terms of the set K-cover problem (also known as the target coverage problem) or as the MDDS problem. Modeling the problem as a K-cover problem was performed for the first time in [13]. In the same work, the problem was shown to be NP-hard. In this context, note that the set K-cover problem is defined on the basis of a bipartite graph in which the first set of nodes are the sensor devices and the second set of nodes are the sensing targets. The aim of the problem is to partition the sensor devices into a maximum number of disjoint sets, with each one covering all targets. As mentioned before, these disjoint sets are then activated one after the other in order to keep the network alive for the maximum period of time. Due to the NP-hardness of the problem, a range of approximate algorithms have been proposed in the literature in order to solve it. Examples, which also include algorithms for closely related problem formulations, are a greedy heuristic [13], some memetic algorithms [14,15,16], a cuckoo search approach [17], and finally, a genetic algorithm [18].

As already indicated above, the problem of maximizing sensor network lifetime is also frequently modeled as an MDDS problem, the goal of which is to identify a partition of the sensor devices into a maximum number of disjoint dominating sets of the corresponding communication graph. The MDDS problem, which belongs to the important family of dominating set problems [19,20,21], was shown to be NP-hard for general graphs [22]. Cardei et al. [23] proved the NP-completeness of a special case of the MDDS problem known as the 3-disjoint dominating sets problem. This variant deals with the question of whether or not a given graph contains three disjoint dominating sets. Nguyen and Huynh [9] proved that the 3-disjoint dominating sets problem remains NP-complete even for the special cases of planar unit disk graphs. Moreover, they introduced and evaluated the performance of four greedy heuristics for the general MDDS problem. In [23], it was also proved that unless P = NP, the MDDS problem has no polynomial-time approximation with a performance guarantee better than 1.5. Finally, the same authors introduced a graph coloring-based heuristic approach. Next, Feige et al. [24] showed that any graph with *n* nodes, a maximum degree of Δ, and a minimum degree of δ has a domatic partition with a size of (1−o(1))(δ+1)/lnΔ. Note that the term o(1) tends to zero with increasing *n*. Moreover, the same authors were able to show the non-existence of an approximation algorithm with an approximation ratio of (1+o(1))lnn unless $NP\subseteq DTIME(n^{O(\log\log n)})$. Finally, they also introduced a centralized algorithm generating a domatic partition with a size of Ω(δ/lnΔ). Moscibroda and Wattenhöfer [25] regarded the MDDS problem as one with a maximizing cluster lifetime. They introduced a randomized, distributed algorithm having—with high probability—a performance ratio of O(log(n)). Finally, a greedy heuristic for the MDDS problem, with a time complexity of $O(n^{3})$, was described in [26].

### 1.3. Existing Work for the MWDDS Problem and Our Contribution

Recently, a weighted variant of the MDDS problem, in which the weights of the nodes of a given undirected graph, G=(V,E), indicate the remaining lifetime of single sensor devices, was introduced in [27]. The authors labeled this problem as the *maximum weighted disjoint dominating sets* (MWDDS) problem. The lifetime of a dominating set in *G* is hereby defined as the minimum of the lifetimes of the nodes that form part of the set. The MWDDS problem asks to identify a domatic partition that maximizes the sum of the lifetimes of the corresponding dominating sets.

In addition, three algorithms based on a local search were provided in [27]. Each of these local search methods takes a solution generated by a greedy heuristic from [26] as the initial solution. The proposed local search methods make use of swap neighborhoods, trying, for example, to exchange nodes between different dominating sets and to incorporate nodes not belonging to any dominating set of the current solution. The three local search methods differ in the type of swaps that are considered. The current state-of-the-art approach for the MWDDS problem is, surprisingly, a greedy heuristic that was introduced in [28]. This algorithm generates one dominating set after the other by adding one node at a time to the partial dominating set under construction.

Given that the greedy heuristic from [28] seems to be very powerful, in this work, we propose a metaheuristic extension of this greedy heuristic. More specifically, we propose a population-based iterated greedy (PBIG) algorithm on the lines of [29,30]. Just as with any other iterated greedy (IG) approach [31], our algorithm iteratively generates a new solution to the problem by partially destroying incumbent solutions and re-constructing the obtained partial solutions by means of a randomized greedy technique. As we will show in the section on the experimental results, our proposed approach clearly outperforms all existing algorithms for the MWDDS problem. In addition to 640 problem instances from the related literature, we likewise evaluate our algorithm—in comparison to the competitors—on 300 random geometric graphs.

### 1.4. Paper Organization

The remainder of this paper is organized as follows. The MWDDS problem is formally introduced, together with notations and basic definitions, in Section 2. This also includes a graphical example and an ILP model for the MWDDS problem. In Section 3, we introduce our algorithmic proposal, a population-based iterated greedy algorithm for solving the MWDDS problem. Finally, Section 4 presents a comprehensive experimental evaluation and a comparison to the current state of the art, while Section 5 summarizes our paper and offers directions for future lines of work.

## 2. The MWDDS Problem

Let G=(V,E,lifetime) be an undirected, node-weighted graph. As already mentioned before, *V* refers to the set of nodes, while E⊆V×V is the set of edges. Moreover, $\mathrm{lifetime}:V\to R^{+}$ is a weight function defined over the set of nodes, assigning a positive weight, lifetime(v)>0, to each node, v∈V. The maximum weighted disjoint dominating sets (MWDDS) problem is then defined, such that any domatic partition D of *G* is a valid solution. The objective function value of a valid solution, $D=\{D_{1},\ldots ,D_{\left|D\right|}\}$, is defined as follows:

(1) $$f\left(D\right):=\sum_{i=1}^{\left|D\right|}\min\left\{\mathrm{lifetime}\left(v\right)|v\in D_{i}\right\}$$

In other words, the quality of a subset, $D_{i}$, is determined as the minimum lifetime of all its nodes. The objective is to find a valid solution, $D^{*}$, that maximizes objective function *f*.

### 2.1. Graphical Example

**Figure 1:**
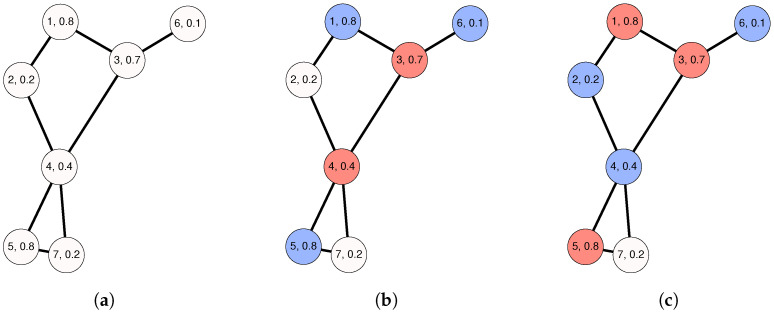
An illustrative example of the MWDDS problem. (**a**) Problem instance; (**b**) Feasible solution D={{3,4},{1,5,6}}, with f(D) = 0.5; (**c**) Optimal solution $D^{*}=\left\{\left\{1,3,5\right\},\left\{2,4,6\right\}\right\}$, with $f(D^{*})=0.8$.

Figure 1 shows a graphical example of the MWDDS problem. In particular, Figure 1a shows an undirected graph on seven nodes. The labels printed within the nodes have the format x,y, where *x* is the nodes’ ID and *y* is the nodes’ lifetime. These lifetime values are normalized to the range [0,1] for the sake of simplicity. Furthermore, the graphic in Figure 1b shows a feasible solution, $D:=\{D_{1}=\left\{3,4\right\},D_{2}=\left\{1,5,6\right\}\}$, which consists of two dominating sets, $D_{1}=\left\{3,4\right\}$ and $D_{2}=\left\{1,5,6\right\}$. According to the node labels, the remaining lifetimes of nodes 3 and 4 are 0.7 and 0.4, respectively. Correspondingly, $D_{1}$ has a lifetime of 0.4. Next, it can also be easily calculated that the lifetime of $D_{2}$ is 0.1 because the individual lifetimes of nodes 1, 5, and 6 are 0.8, 0.8, and 0.1, respectively. The objective function value f(D) of *D* is calculated as the sum of the lifetimes of the dominating sets in *D*. Therefore, the lifetime of *D* is 0.5. Finally, the graphic in Figure 1c demonstrates the optimal solution, $D^{*}:=\left\{D_{1}=\left\{1,3,5\right\},D_{2}=\left\{2,4,6\right\}\right\}$, to this problem instance. Hence, the lifetime of $D_{1}$ is 0.7, while the lifetime of $D_{2}$ is 0.1. Therefore, the objective function value $f(D^{*})$ of $D^{*}$ is 0.8. Since this small sample graph contains a node with a degree of 1, any valid solution can contain at most two disjoint dominating sets.

### 2.2. ILP Model for the MWDDS Problem

The following integer linear programming (ILP) model for the MWDDS problem was introduced in [28]. First, we describe the sets of variables and their domains utilized by this model:

1. A binary variable, $x_{ij}$, for each combination of a node, $v_{i}$ (i=1,…,n), and a possible disjoint set, $D_{j}$ (j=1,…,δ(G)+1), indicates whether or not node $v_{i}$ forms part of the dominating set $D_{j}$. That is, when $x_{ij}=1$, node $v_{i}$ is assigned to the dominating set $D_{j}$. In this context, remember that (1) δ(G):=min{deg(v)∣v∈V}, and (2) the number of disjoint dominating sets in a graph is bounded from above by δ(G)+1.
2. Second, a binary variable, $y_{j}$ (j=1,…,δ(G)+1), indicates whether the j—th dominating set is utilized at all.
3. Finally, a real-valued variable, $z_{j}\in \left[0,M\right]$, is used to store the weight of the j—th dominating set. In our implementation of the model, we used M:=max{lifetime(v)∣v∈V}.

The MWDDS can then be stated in terms of an ILP model in the following way:

(2)max∑j=1δ(G)+1zj(3)s.t.∑j=1δ(G)+1xij≤1i=1,…,n(4)∑vk∈N(vi)xkj≥yj−xijj=1,…,δ(G)+1(5)yj≥xiji=1,…,n and j=1,…,δ(G)+1(6)xij·lifetime(vi)+(1−xij)·M≥zji=1,…,n and j=1,…,δ(G)+1(7)yj·M≥zjj=1,…,δ(G)+1(8)yj≥yj+1j=1,…,δ(G)(9)zj≥zj+1j=1,…,δ(G)

The objective function is the sum of the weight values of all dominating sets. Constraints (3) ensure that each node is assigned to one dominating set at most. In this way, the chosen dominating sets are disjoint. Next, constraints (4) are the usual dominating set constraints, that is, they make sure that the set of nodes assigned to the *j*-th set (if utilized) form a dominating set of *G*. Furthermore, constraints (5) make sure that nodes can only be assigned to utilized dominating sets. Constraints (6) correctly determine the lifetimes of the utilized dominating sets. This is accomplished by setting the value of the variable $z_{j}$ of the *j*-th dominating set (if utilized) to the minimum of the lifetime values of all nodes assigned to it. Next, note that the objective function (Equation (2)) is only correct if the weight value of the dominating sets not utilized is set to zero. This is ensured by constraints (7). The remaining two sets of constraints, (8) and (9), are not required for the correctness of the ILP. They were introduced for tie-breaking purposes that have the following effect: (1) if *k* dominating sets are utilized, they are assigned to sets 1,…,k, and (2) the utilized dominating sets are ordered according to a non-increasing weight value.

## 3. Proposed Algorithm

Our population-based iterated greedy (PBIG) algorithm is a population-based extension of the well-known iterated greedy (IG) metaheuristic [31], that is, it produces a sequence of solutions by iterating over a constructive greedy heuristic in the following way. At each iteration, first, some part of the current/incumbent solution is removed, and second, the greedy heuristic is applied to the resulting partial solution in order to again obtain a complete solution. The first of these phases is called the *destruction phase*, while the second one is known as the *reconstruction phase*. A high-level description of our PBIG algorithm for solving the MWDDS problem is given in Algorithm 1. Apart from a problem instance, PBIG requires seven input parameters: (1) the population size ($p_{\mathrm{size}}$), (2) the lower bound of the degree of greediness during solution construction ($\det_{\min}$), (3) the upper bound of the degree of greediness during solution construction ($\det_{\max}$), (4) the lower bound of the degree of solution destruction (${\mathrm{destr}}_{\min}$), (5) the upper bound of the degree of solution destruction (${\mathrm{destr}}_{\max}$), (6) the maximum number of iterations without the improvement of the best-so-far solution $D_{\mathrm{bsf}}$ before applying a restart ($\max_{\mathrm{noimpr}}$), and (7) the degree of partial solution removal ($r_{\mathrm{del}}$). Moreover, note that in our algorithm, each solution D has two solution-specific parameters: the individual destruction rate, ${\mathrm{destr}}_{D}$, and the individual degree of greediness, $\det_{D}$. The use of all parameters will be carefully described below.
**Algorithm 1** PBIG for the MWDDS problem.**Input:** A problem instance G=(V,E,lifetime) and values for parameters $p_{\mathrm{size}}$, ${\mathrm{destr}}_{\min}$, ${\mathrm{destr}}_{\max}$, $\det_{\min}$, $\det_{\max}$, $\max_{\mathrm{noimpr}}$, and $r_{\mathrm{del}}$.**Output:** A family of disjoint dominating sets $D=\{D_{1},D_{2},\cdots ,D_{k}\}$1:P:=GenerateInitialPopulation()2:$D_{\mathrm{bsf}}:=\mathrm{argmmax}\left\{f\left(D\right)|D\in P\right\}$3:cnt:=04:**while** termination condition not satisfied **do**5:   $P_{\mathrm{new}}:=\emptyset$6:   **for** each candidate solution D∈P **do**7:     $D^{p}:=\mathrm{DestroyPartially}\left(D\right)$8:     $\hat{D}:=\mathrm{GreedyMWDDS}\left(D^{p}\right)$ // see Algorithm 29:     $P_{\mathrm{new}}\leftarrow P_{\mathrm{new}}\cup \left\{\hat{D}\right\}$10:     $\mathrm{AdaptParameters}(D,\hat{D})$11:   **end for**12:   $D_{\mathrm{ib}}:=\mathrm{argmmax}\left\{f\left(D\right)|D\in P_{\mathrm{new}}\right\}$13:   **if** $f\left(D_{\mathrm{ib}}\right)>f\left(D_{\mathrm{bsf}}\right)$ **then** $D_{\mathrm{bsf}}:=D_{\mathrm{ib}}$, cnt:=0 **else** cnt:=cnt+1 **end if**14:   P←SelectNextPopulation$\left(P,P_{new},cnt\right)$15:**end while**16:**return**$D_{\mathrm{bsf}}$

The algorithm works as follows. A set of $p_{\mathrm{size}}$ solutions for the initial population are generated in the function GenerateInitialPopulation() (see line 1 of Algorithm 1). Moreover, the best-so-far solution, $D_{\mathrm{bsf}}$, and the cnt counter are initialized. Afterwards, the main loop of the algorithm starts. Each iteration consists of the following steps. Each solution, D∈P, is partially destroyed using procedure DestroyPartially(D) (see line 7 of Algorithm 1), resulting in a partial solution, $D^{p}$. On the basis of $D^{p}$, a complete solution, $\hat{D}$, is then constructed using the procedure $\mathrm{GreedyMWDDS}(D^{p})$ (see line 8 of Algorithm 1). Each newly obtained complete solution is stored in an initially empty, new population, $P_{new}$. Moreover, the individual destruction rates and the individual degree of greediness of D and $\hat{D}$ are adapted in the function, $\mathrm{AdaptParameters}(D,\hat{D})$. Then, the iteration-best solution, $D_{\mathrm{ib}}$, is determined, and in case this solution improves over $D_{\mathrm{bsf}}$, the non-improvement counter cnt is set back to zero. Otherwise, this counter is incremented. As a last step in each iteration procedure, $\mathrm{SelectNextPopulation}(P,P_{new},cnt)$ chooses the solutions for the population of the next iteration, maintaining the population size constant at $p_{\mathrm{size}}$ at all times. Finally, the algorithm terminates when a given CPU time limit has been reached, and the best found solution is returned. The five procedures mentioned above are described in more detail below.

$\mathrm{GenerateInitialPopulation}(p_{\mathrm{size}})$: Each of the $p_{\mathrm{size}}$ solutions of the initial population is constructed by applying the procedure GreedyMWDDS(·), with the empty solution $D_{0}=\emptyset$ as input. Note that this procedure depends on the degree of greediness, which is the same for all solutions because each empty partial solution, $D_{0}$, is initialized with $\det_{D_{0}}=\det_{\max}$, that is, the upper bound for the greediness of solution construction. Moreover, it is also initialized with ${\mathrm{destr}}_{D_{0}}={\mathrm{destr}}_{\min}$, that is, the lower bound of the destruction rate is set as the initial value.

$\mathrm{GreedyMWDDS}(D^{p})$: The reconstruction procedure follows the general principle of a greedy algorithm, which builds a complete solution step-by-step, selecting one additional node at each construction step. In this work, we adopt the recent greedy heuristic presented in [28]. However, we extend this greedy heuristic (1) in order to allow for randomized steps and (2) to be able to take a partial (possibly non-empty) solution as input. In other words, our randomized greedy mechanism takes as input a partial solution, $D^{p}$, which might be empty. Note that such a partial solution is composed of independent, partially destroyed dominating sets. Now, the construction of a complete solution, $D=\{D_{1},D_{2},\cdots ,D_{m}\}$, on the basis of $D^{p}=\left\{D_{1}^{p},D_{2}^{p},\cdots ,D_{k}^{p}\right\}$ (where k≤m) is performed by consecutively dealing with the generation of $D_{i}$ starting from $D_{i}^{p}$ for all i=1,…,m. In this process, whenever $i>|D^{p}|$ or $D^{p}=\emptyset$, $D_{i}$ is initialized with the empty set. In the following, $V_{\mathrm{rem}}$ denotes the set that includes all nodes that are not yet added to a dominating set of a current (partial) solution $D^{p}$. That is, when receiving a partial solution $D^{p}$ as input, $V_{\mathrm{rem}}:=V\backslash {⋃}_{i=1}^{|D^{p}|}D_{i}^{p}$. Thus, if $D^{p}=\emptyset$, then $V_{\mathrm{rem}}:=V$.

In the following, we describe the way to obtain a dominating set, $D_{i}$, starting from a partial dominating set, $D_{i}^{p}$ (possibly being empty). At the start, all nodes in *V* can be divided into three disjoint subsets with respect to $D_{i}$:

- *Black nodes*: node from $D_{i}$;
- *Gray nodes*: nodes that are not in $D_{i}$ but are dominated by black nodes, that is, all nodes in $N\left(D_{i}\right)\backslash D_{i}$, where $N\left(D_{i}\right):={⋃}_{v\in D_{i}}N\left(v\right)$ and N(v) is the neighborhood of *v* in *G*;
- *White nodes*: all nodes from *V* that are neither black nor gray.

With this classification of the nodes, we can define the *white degree* of a node, $v\in V_{\mathrm{rem}}$—with respect to $D_{i}$—as the number of white nodes from the closed neighborhood of *v*:

(10) $$\mathrm{white}\_\mathrm{degree}\left(v\right):=|\{u\in N\left[v\right]\cap V_{\mathrm{rem}}|u\;\mathrm{is}\;a\;\mathrm{white}\;\mathrm{node}\;\mathrm{with}\;\mathrm{respect}\;\mathrm{to}\;D_{i}\}|$$

To be able to choose the next node to be added to set $D_{i}$ at the current construction step, all nodes from $V_{\mathrm{rem}}$ are evaluated using a greedy function denoted by score(·), which is calculated as follows:

(11) $$\mathrm{score}\left(v\right):=\mathrm{lifetime}\left(v\right)*white\_degree\left(v\right)\;\forall v\in V_{\mathrm{rem}}$$

Then, the randomization incorporated in our greedy heuristic is implemented using a quality-based restricted candidate list (RCL). The size of the RCL is controlled by the solution-specific parameter, $\det_{D}\in \left[0,1\right]$, called the degree of greediness. Its value is adaptive and depends on the quality of the generated solution, as explained further below.

Let ${\mathrm{score}}_{min}:=\min\left\{\mathrm{score}\left(v\right)|v\in V_{\mathrm{rem}}\right\}$ and ${\mathrm{score}}_{max}:=\max\left\{\mathrm{score}\left(v\right)|v\in V_{\mathrm{rem}}\right\}$. The RCL then contains all nodes, $v\in V_{\mathrm{rem}}$, whose scoring value is greater than or equal to ${\mathrm{score}}_{min}+\det_{D}\left({\mathrm{score}}_{max}-{\mathrm{score}}_{min}\right)$. Note that when $\det_{D}=1$, our solution construction procedure behaves similar to a deterministic greedy heuristic. On the other hand, setting $\det_{D}=0$ leads to pure random construction. Finally, a node is selected at random from the RCL to be incorporated into the partial dominating set, $D_{i}$.

Once $D_{i}$ is a dominating set, it might contain redundant nodes which—after identification—can be safely removed. In this context, note that a node is redundant if and only if any node from its closed neighborhood is dominated by at least two nodes from $D_{i}$. If, by removing redundant nodes, the node with the lowest lifetime in $D_{i}$ can be removed, the overall lifetime of $D_{i}$ is improved. After removing redundant nodes, $D_{i}$ is placed in the solution, D, under construction. Afterwards, the set $V_{\mathrm{rem}}$ is updated accordingly before moving to the construction of the next dominating set, $D_{i+1}$. This solution construction process ends once no further dominating set can be generated from the nodes in $V_{\mathrm{rem}}$. This occurs when it is impossible to complete the partial dominating set under construction because either (1) $V_{\mathrm{rem}}$ is empty or (2) no node from $V_{\mathrm{rem}}$ has a white closed neighbor. The pseudo-code of the complete procedure is shown in Algorithm 2.

DestroyPartially(D): Let $D=\{D_{1},D_{2},\cdots ,D_{m}\}$ be the valid solution given as input to the destruction procedure. In the following, we outline the three strategies for destruction that are conducted sequentially: partial solution removal, worst node removal, and random node removal. In this context, it is important to note that, on the one hand, the best solution does not necessarily correspond to the solution with the maximum number of disjoint dominating sets; on the other hand, the size of a re-constructed solution after performing the destruction and reconstruction phases may be different to the size of the solution that served as input to these two phases. With this in mind, some disjoint dominating sets should be completely removed as a first step of partial solution destruction. For this purpose, the $\max\{1,\lfloor r_{\mathrm{del}}\cdot \left|D|\rfloor \right\}$ randomly chosen dominating sets are removed from D, resulting in a partial solution $D^{p}=\left\{D_{1}^{p},D_{2}^{p},\cdots ,D_{r}^{p}\right\}$, where r<m.

Then, since the quality of a subset, $D_{i}\in D$, is determined as the minimum lifetime of all its nodes, keeping the node with the smallest lifetime during the partial destruction makes its further improvement impossible. For this reason, the removal of the node, $v^{worst}:=\mathrm{argmin}\left\{\mathrm{lifetime}\left(v\right)|v\in D_{i}^{p}\right\}$, from each subset, $D_{i}^{p}\in D^{p}$, i=1,⋯,r, becomes necessary. Afterwards, a set of $\left\lfloor {\mathrm{destr}}_{D}\times |D_{i}^{p}|\right\rfloor$ randomly chosen nodes are removed from each subset, $D_{i}^{p}\in D^{p}$, in an iterative way. Thus, at each step, exactly one randomly chosen vertex is removed.
**Algorithm 2** Procedure $\mathrm{GreedyMWDDS}(D^{P})$.**Input:** A (possibly empty) partial solution $D^{p}=\left\{D_{1}^{p},D_{2}^{p},\cdots ,D_{k}^{p}\right\}$.**Output:** A complete valid solution $D=\{D_{1},D_{2},\cdots ,D_{m}\}$1:D:=∅2:**if**$D^{P}=\emptyset$, **then**$V_{\mathrm{rem}}:=V$**else**$V_{\mathrm{rem}}:=V\backslash {⋃}_{i=1}^{|D^{p}|}D_{i}^{p}$3:stopping_condition:= false4:i:=05:**while** not stopping_condition **do**6:   i:=i+17:   **for** each node v∈V **do**8:     color(v):= WHITE9:   **end for**10:   **if** ($D^{P}=\emptyset$ **or** $i>|D^{P}|$), **then** $D_{i}:=\emptyset$ **else** $D_{i}:=D_{i}^{p}$11:   **while** $D_{i}$ is not a dominating set of *G* (that is, $N[D_{i}]\neq V$) **and** not a stopping_condition **do**12:     **if** $V_{\mathrm{rem}}=\emptyset$ **then**13:        stopping_condition:= true14:     **else**15:        ${\mathrm{score}}_{max}:=\max\left\{\mathrm{score}\left(v\right)|v\in V_{\mathrm{rem}}\right\}$16:        **if** ${\mathrm{score}}_{max}=0$ **then**17:          stopping_condition:= true {▸ No node from $V_{\mathrm{rem}}$ has a white closed neighbor}18:        **else**19:          ${\mathrm{score}}_{min}:=\min\left\{\mathrm{score}\left(v\right)|v\in V_{\mathrm{rem}}\right\}$20:          RCL $:=\{v\in V_{\mathrm{rem}}|\mathrm{score}\left(v\right)\geq {\mathrm{score}}_{min}+\det_{D}\left({\mathrm{score}}_{max}-{\mathrm{score}}_{min}\right)\}$21:          Choose $v^{*}$ uniformly at random from RCL22:          $D_{i}:=D_{i}\cup \left\{v^{*}\right\}$23:          $V_{\mathrm{rem}}:=V_{\mathrm{rem}}\backslash \left\{v^{*}\right\}$24:          **for** each node $u\in N(v^{*})$ **do**25:             **if** ( color(u)= WHITE ) **then**26:               color(v):= GRAY27:             **end if**28:          **end for**29:          $color(v^{*}):=$ BLACK30:        **end if**31:     **end if**32:   **end while**33:   **if** not stopping_condition **then**34:     $\mathrm{Reduce}(D_{i}$, $V_{\mathrm{rem}}$) {▸ Remove redundant nodes}35:     $D:=D\cup \{D_{i}\}$36:   **end if**37:**end while**38:**return**D.

$\mathrm{AdaptParameters}(D,\hat{D})$: The solution-specific parameters—concerning the degree of greediness (RCL parameter) and the destruction rate—are adapted in relation to the results of the destruct and re-construct procedures. More specifically, while the newly generated solution, $\hat{D}$, is initialized with the default values—that is, $\det_{\hat{D}}:=\det_{\max}$ and ${\mathrm{destr}}_{\hat{D}}:={\mathrm{destr}}_{\min}$—the adaptation of the parameter values of D depends on $\hat{D}$, and vice versa. In case $f\left(\hat{D}\right)>f\left(D\right)$, $\hat{D}$ will adopt the values of D, that is, $\det_{\hat{D}}:=\det_{D}$ and ${\mathrm{destr}}_{\hat{D}}:={\mathrm{destr}}_{D}$. Otherwise, the values of D are adapted as follows:

(12) $$\begin{array}{ccc} \det_{D} & := & \det_{D}-0.1 \end{array}$$

(13) $$\begin{array}{ccc} {\mathrm{destr}}_{D} & := & {\mathrm{destr}}_{D}+\frac{{\mathrm{destr}}_{\max}-{\mathrm{destr}}_{\min}}{9} \end{array}$$

Once the value of $\det_{D}$ falls below the lower bound, $\det_{\min}$, it is set back to the upper bound, $\det_{\max}$. In the same way, once the value of ${\mathrm{destr}}_{D}$ exceeds the upper bound, ${\mathrm{destr}}_{\max}$, it is set back to the lower bound, ${\mathrm{destr}}_{\min}$.

Note that that the constants 0.1 and 9 were fixed after preliminary experiments. In contrast, the values of seven important parameters will be determined by scientific tuning experiments (see Section 4.2). The denominator in the case of the adaptation of ${\mathrm{destr}}_{D}$ was set to 9 in order to have 10 different values between ${\mathrm{destr}}_{\min}$ and ${\mathrm{destr}}_{\max}$. The motivation behind this adaptive scheme for the degree of greediness and the destruction rate is to use a higher degree of greediness and a smaller solution destruction as this leads to better solutions, and to move towards a lower degree of greediness and a higher destruction once no more improving solutions are found.

$\mathrm{SelectNextPopulation}(P,P_{\mathrm{new}},cnt)$: This last function concerns the selection/generation of the solutions for the population of the next iteration. If $cnt<\max_{\mathrm{noimpr}}$, the new population, P, is simply filled with the $p_{\mathrm{size}}$ best solutions from $P\cup P_{\mathrm{new}}$. In case $cnt=\max_{\mathrm{noimpr}}$, all solutions, apart from the best one, are deleted from P, and $p_{\mathrm{size}}-1$ new solutions are added via the use of the GreedyMWDDS(D) procedure (used with D=∅ as input). Note that in this case, the RCL parameter, $\det_{D}$, is each time randomly picked from {0.5,0.6,0.7,0.8,0.9,1}. This set of values was chosen after preliminary experiments. The variation in this set potentially ensures some diversification in the search space, with the hope of covering unexplored areas of the search space. Moreover, cnt is set to zero. In summary, this function implements a restart procedure which is performed once $\max_{\mathrm{noimpr}}$ iterations have been performed without the improvement of the best-so-far solution, $S_{\mathrm{bsf}}$.

## 4. Experimental Evaluation

We implemented the proposed PBIG algorithm in ANSI C++ using GCC 10.2.0 for the compilation of the software. Moreover, we decided to compare PBIG with the following algorithmic approaches: (1) the best of three available local search approaches from the literature, called VD (Variable Depth) [27]; (2) our own greedy heuristic, labeled GH-MWDDS${}^{+}$ [28]; (3) application of the ILP solver ILOG CPLEX 20.01 in a single-threaded mode to all problem instances. Note that, surprisingly, GH-MWDDS${}^{+}$ is currently the state-of-the-art method used to solve the MWDDS problem, outperforming both the local search method (VD) and CPLEX. In order to conduct a fair comparison to the VD algorithm, we used the original source code provided by the authors of [27].

The time limit for each application of CPLEX was set to 2 CPU hours. Moreover, the experiments were performed on a cluster of machines with two Intel^®^ Xeon^®^ Silver 4210 CPUs, with 10 cores of 2.20 GHz and 92 Gbytes of RAM.

### 4.1. Problem Instances

All considered techniques were applied to two sets of benchmark instances. The first set, consisting of 640 random graph instances, was already used for the evaluation of GH-MWDDS${}^{+}$ in [28]. In particular, this set—henceforth called Set1—contained graphs with n∈{50,100,150,200,250} nodes. For each value of *n*, there were graphs of different densities, expressed by the average degree, *d*. In the case of n=50, for example, Set1 contained graphs with average degrees of d∈{15,20,25,30,35}. For each combination of *n* and *d*, Set1 contained 20 randomly generated graphs.

In order to test our algorithms on graphs that are also commonly used to model sensor networks, we generated an additional set of benchmark instances (Set2) consisting of random geometric graphs (RGGs). These graphs were generated by scattering n∈{100,500,1000} nodes randomly on the square, ${\left[0,1\right]}^{2}$. This means that each node, *i*, had its location in $\left(x_{i},y_{i}\right)\in {\left[0,1\right]}^{2}$. Two nodes, *i* and *j*, were then connected by an edge if and only if the Euclidean distance between *i* and *j* was smaller or equal to a predefined threshold value, r>0. For each n∈{100,500,1000}, we considered five different threshold values. Moreover, for each combination of *n* and *r*, we randomly generated 20 graphs. Accordingly, Set2 consists of 300 problem instances.

Finally, note that—both in the case of Set1 and Set2—each node (sensor) of the network was given a random real value between 0 and 1 as node weight (lifetime). Both benchmark sets can be obtained at https://www.iiia.csic.es/~christian.blum/research.html#Instances (accessed on 16 January 2022).

### 4.2. Algorithm Tuning

PBIG requires well-working parameter values for the following seven parameters:

1. Population size ($p_{\mathrm{size}}$);
2. Lower bound of the determinism rate ($\det_{\min}$);
3. Upper bound of the determinism rate ($\det_{\max}$);
4. Lower bound of the destruction rate (${\mathrm{destr}}_{\min}$);
5. Upper bound of the destruction rate (${\mathrm{destr}}_{\max}$);
6. Number of iterations without improvement ($\max_{\mathrm{noimpr}}$);
7. Deletion rate ($r_{\mathrm{del}}$).

For the the purpose of parameter tuning, we used the scientific tuning software irace [32]. More specifically, we tuned PBIG separately for Set1 and Set2. For this purpose, we generated specific tuning instances as follows. For Set1, exactly one instance was randomly generated for the following combinations of *n* (number of nodes) and *d* (average degree): (n=50,d=15), (n=50,d=35), (n=100,d=20), (n=100,d=60), (n=150,d=30), (n=150,d=90), (n=200,d=40), (n=200,d=100), (n=250,d=50), and (n=250,d=140). In other words, 10 instances were specifically generated in order to tune PBIG for its application to instances from Set1. In this case, the irace software was run with a budget of 5000 algorithm applications. Regarding Set2, we randomly generated one tuning instance for each of the following combinations of *n* and *r* (threshold value for connecting two nodes): (n=100,r=0.2), (n=100,r=0.3), (n=500,r=0.1), (n=500,r=0.2), (n=1000,r=0.05), and (n=1000,r=0.15). In this case, as irace is applied with only six tuning instances, the budget was limited to 3000 algorithm applications. The obtained parameter value settings are shown in Table 1.

### 4.3. Results and Discussion

First of all, note that PBIG was applied exactly once to each problem instance, with a computation time limit of n/2 CPU seconds. Table 2 presents the results of all competing methods—that is, CPLEX, VD, GH-MWDDS${}^{+}$, and PBIG—for the instances of Set1. The first two columns indicate the problem instance type in terms of: (1) the number of nodes (*n*) and (2) the average degree (*d*). Naturally, the density of the networks grows with an increasing average degree, *d*. Each table row provides the average result of each competing algorithm for the 20 generated problem instances concerning the corresponding combination of *n* and *d*. Table columns 3 and 4 show the results of CPLEX. The first of these columns (with the heading “Value”) provides the average quality of the best solutions generated by CPLEX for 20 instances, while the second column presents the average gap (in percent) between the objective function value of the solutions obtained by CPLEX and the best upper bounds identified by CPLEX. The results of the other three competitors are shown by the columns with the headings “Value” and “Time”. The first column provides—as in the case of CPLEX—the average quality of the generated solutions, while the second one provides the computation time. In the case of VD and GH-MWDDS${}^{+}$, the computation time corresponds to the time at which the algorithm terminated, while in the case of PBIG, the computation time is the time at which the best solution of a run was found on average. The corresponding standard deviation in the case of PBIG is provided in an additional column with the heading “$\sigma_{Time}$”. Finally, note that the best result in each row is indicated in bold font.

The results displayed in Table 2 allow for the following observations:

- As already mentioned in [28], solving the MWDDS problem by means of an ILP solver such as CPLEX is only useful in the context of the smallest of all problem instances. In fact, even though CPLEX obtains the best results in the case of (n=50,d=15), the gap information indicates that—even in this case—CPLEX is far from being able to prove optimality.
- For all instances, apart from (n=50,d=15), PBIG outperforms the remaining approaches. In particular, the current state-of-the-art method, GH-MWDDS${}^{+}$, is consistently outperformed. This shows that our way of extending the solution construction mechanism of GH-MWDDS${}^{+}$ into a PBIG algorithm was successful.
- Both GH-MWDDS${}^{+}$ and PBIG clearly outperform the best local search algorithm (VD) from the literature. In fact, while VD achieves an average solution quality of 1.757, GH-MWDDS${}^{+}$ obtains an average solution quality of 9.515, and PBIG achieves one of 10.321. This does not only hold for solution quality but also for computation time. While VD requires a computation time of 984.143 seconds on average, GH-MWDDS${}^{+}$ requires only 0.006 seconds. Even the average computation time of PBIG is, with 20.125 seconds, around 50 times lower than that of VD.

Next, we study the results obtained by CPLEX, GH-MWDDS${}^{+}$, and PBIG for the new RGG instances from Set2. These results are shown in Table 3, which has the same structure as Table 2. The only exception is the second table column, which provides the threshold value, *r*, used to generate the RGGs, instead of the average degree, *d*. The following conclusions can be drawn based on the obtained results:

- First of all, CPLEX does seem to have fewer problems in solving RGG instances in comparison to random graphs. In fact, CPLEX is able to solve all 60 instances with n=100 and r∈{0.2,0.225,0.25} to proven optimality. Furthermore, 19 out of 20 instances with n=100 and r=0.275 are solved to optimality, as well as 18 out of 20 cases with n=100 and r=0.3. This is in contrast to the case of RGs, for which CPLEX was not even able to solve problem instances with 50 nodes to optimality. Nevertheless, for the larger instances (with n∈{500,1000}) CPLEX was, with very few exceptions, only able to derive the trivial solutions that do not contain any dominating sets.
- PBIG obtains the same results as CPLEX in those cases in which CPLEX is able to provide optimal solutions. Moreover, PBIG is able to do so in very short computation times of less than 10 s.
- As in the case of the instances in Set1, PBIG also consistently outperforms the other competitors for the RGG instances in Set2. While GH-MWDDS${}^{+}$ obtains an average solution quality of 2.911, PBIG achieves one of 3.134.

**Figure 2:**
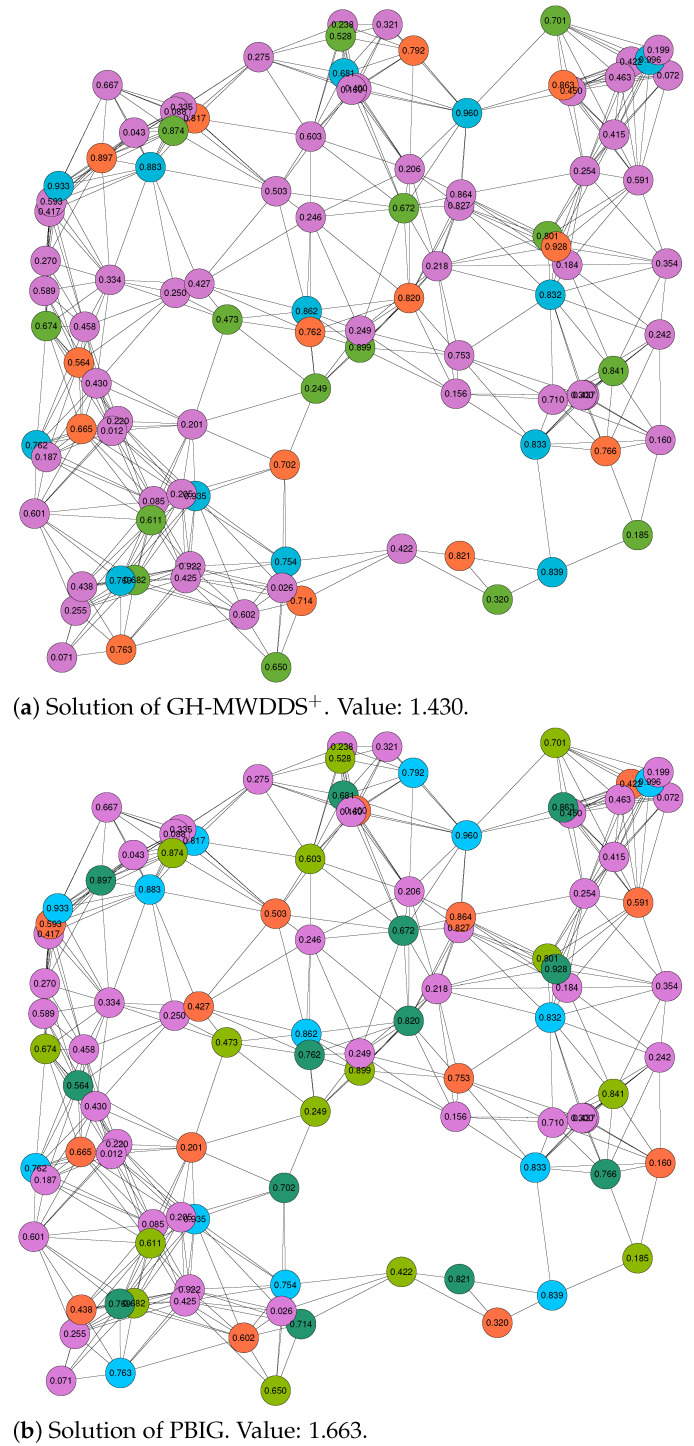
Solutions of GH-MWDDS${}^{+}$ (**a**) and PBIG (**b**) for the first RGG graph with 100 nodes and a threshold value of r=0.2. The lifetime of each node is provided as the node label. Moreover, the node colors indicate to which dominating set a node belongs. In both cases, the color purple indicates that the respective node is not chosen for a dominating set.

**Figure 3:**
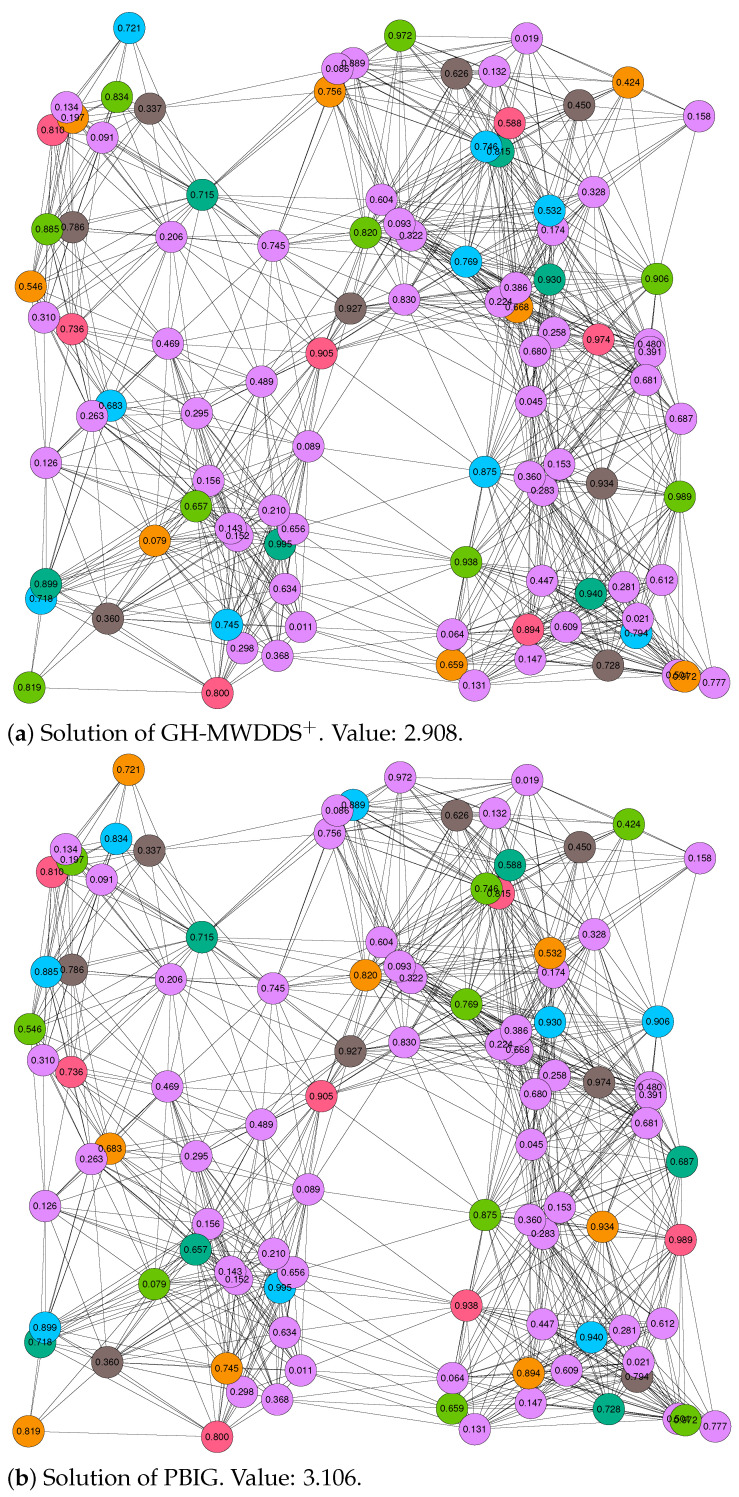
Solutions of GH-MWDDS${}^{+}$ (**a**) and PBIG (**b**) for the first RGG graph with 100 nodes and a threshold value of r=0.3. The lifetime of each node is provided as the node label. Moreover, the node colors indicate to which dominating set a node belongs. In both cases, the color purple indicates that the respective node is not chosen for a dominating set.

Finally, we decided to study the structure of the solutions provided by GH-MWDDS${}^{+}$ and PBIG in more detail. In particular, this was performed for two cases: the first (out of 20) RGG graphs with 100 nodes and a threshold value of r=0.2, and the first (out of 20) RGG graphs with 100 nodes and a threshold value of r=0.3. In both cases, we graphically present the solutions of GH-MWDDS${}^{+}$ and PBIG in Figure 2 (for the graph with r=0.2) and Figure 3 (for the graph with r=0.3). Note that the node color in all the graphics indicates the dominating set to which a node belongs; the purple color indicates that the corresponding node is not assigned to any of the disjoint dominating sets. The four solutions shown in these figures are additionally provided in textual form in Table 4. The four sub-tables provide all disjoint dominating sets, the color in which these sets are shown in the graphics of Figure 2 and Figure 3, the number of nodes contained in all dominating sets, their lifetime, and the lists of nodes belonging to the dominating sets.

The following interesting observations can be made. First, in both cases, the structure of the PBIG solution is quite different to the structure of the GH-MWDDS${}^{+}$ solution. This means that PBIG does not just locally improve the GH-MWDDS${}^{+}$ solutions; it often seems to perform a profound restructuring. Second, in the case of the graph with (n=100,r=0.2), PBIG comes up with a solution that contains one more dominating set than the GH-MWDDS${}^{+}$ solution. This leads to the fact that the PBIG solution leaves less nodes unused in comparison to the GH-MWDDS${}^{+}$ solution (44 nodes unused vs. 58 nodes). Third, the solutions of PBIG and GH-MWDDS${}^{+}$, in the case of the graph with n=100 and r=0.3 (Figure 3), indicates that making use of more nodes does not always lead to better solutions. More specifically, both algorithms generate solutions with six disjoint dominating sets. While the GH-MWDDS${}^{+}$ solution makes use of 47 nodes, the PBIG solution only makes use of 46 nodes. Nevertheless, the PBIG solution is better, with an objective function value of 3.106, in comparison to a quality of 2.908 for the solution generated by GH-MWDDS${}^{+}$. This is because the dominating sets in the PBIG solutions have a longer lifetime on average than those in the GH-MWDDS${}^{+}$ solution.

**Figure 4:**
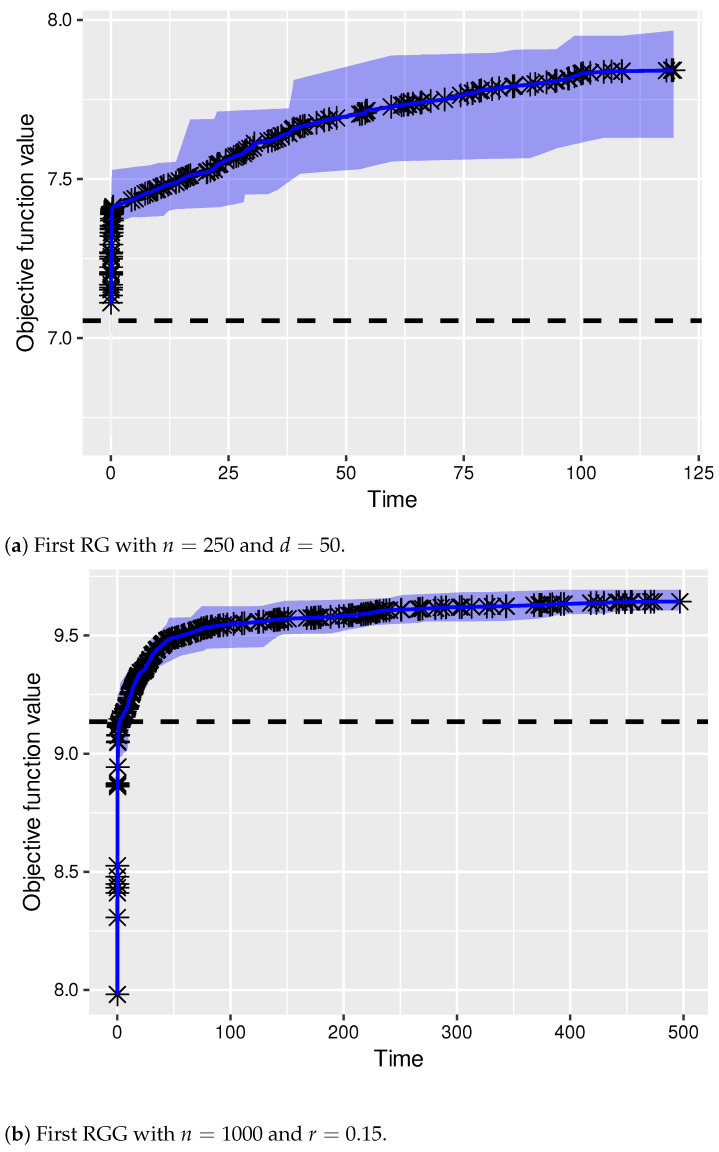
Evolution of the quality of the solutions produced by PBIG over time. Both graphics show the results of 10 independent PBIG runs.

Finally, we decided to show the evolution of the quality of the solutions produced by PBIG over time. This was performed for two of the hardest cases. In particular, we chose the first random graph, with n=250 nodes and an average node degree of d=50, and the first random geometric graph, with n=1000 nodes and a threshold of r=0.15. Remember that the computation time limit is n/2 CPU seconds in both cases. The graphics in Figure 4 show PBIG’s evolution for 10 runs per problem instance. The shaded area around the average behavior line indicates the variance of the algorithm. Moreover, the horizontal, dashed lines indicate the quality of the solutions produced by GH-MWDDS${}^{+}$. Note that in both cases, PBIG outperforms GH-MWDDS${}^{+}$ very early in each run. In the case of Figure 4a, most of the runs even start off with a solution better than the one of GH-MWDDS${}^{+}$. Additionally, note that the given computation time is sufficient in both cases as PBIG shows clear signs of convergence before reaching the computation time limit of 125 CPU seconds in the case of Figure 4, and 500 CPU seconds in the case of Figure 4b.

## 5. Conclusions

This paper dealt with lifetime maximization in wireless sensor networks by means of solving an optimization problem known as the maximum weighted disjoint dominating sets problem. As shown by the weak results of the ILP solver, CPLEX, this problem is a challenging combinatorial optimization problem. In this work, we extended an existing high-quality greedy algorithm from the literature towards a population-based iterated greedy algorithm. This algorithm worked on a population of solutions. At each iteration, it applied partial destruction to each solution in the population. Subsequently, the obtained partial solutions were subjected to re-construction, often resulting in different and improved solutions. We compared our approach to three competitors: the application of CPLEX, the best greedy algorithm from the literature, and the best available local search method. This comparison was based on two benchmark sets. The first set, consisting of 640 random graphs, was taken from the related literature, while the second set, consisting of 300 random geometric graphs, was newly generated for this work. In summary, we can say that our population-based iterated greedy algorithm consistently outperformed all competitors. This algorithm can therefore be called the new state-of-the-art approach for the tackled problem.

Given the weak performance of CPLEX for this problem, one promising line of future work might be that of dealing with the development of specialized exact approaches. Another line of research might focus on hybrid techniques such as construct, merge, solve, and adapt (CMSA) [33]. In particular, CMSA allows users to take profit from ILP solvers such as CPLEX, even in the context of large problem instances for which a direct application of CPLEX is currently not beneficial.

## Figures and Tables

**Table 1:** Parameters, value domains, and values chosen for PBIG by irace.

Parameter	Domain	Value (Set1)	Value (Set2)
$p_{\mathrm{size}}$	{1,…,100}	62	42
$\det_{\min}$	[0,1]	0.91	0.56
$\det_{\max}$	[0,1]	0.96	0.99
${\mathrm{destr}}_{\min}$	[0,1]	0.44	0.22
${\mathrm{destr}}_{\max}$	[0,1]	0.61	0.44
$\max_{\mathrm{noimpr}}$	{1,500}	417	244
$r_{\mathrm{del}}$	[0.1,0.5]	0.11	0.17

**Table 2:** Numerical results for the instances of Set1 (random graphs).

*n*	*d*	CPLEX			VD			GH-MWDDS${}^{+}$			PBIG		
		Value	Gap (%)		Value	Time (s)		Value	Time (s)		Value	Time (s)	$\sigma_{\mathrm{Time}}$
50	15	**2.779**	32.839		0.555	1.984		2.191	0.002		2.716	2.935	6.080
	20	3.922	121.088		1.014	7.651		3.450	0.000		**3.960**	2.763	5.228
	25	5.098	158.038		1.490	4.885		4.672	0.000		**5.244**	2.269	5.475
	30	6.432	200.567		2.780	13.117		6.042	0.000		**6.663**	1.993	2.607
	35	7.929	197.434		3.166	25.048		7.546	0.000		**8.189**	5.615	5.841
100	20	2.467	405.669		0.423	30.458		2.842	0.001		**3.475**	17.670	17.685
	30	3.202	>1000.0		0.576	31.796		4.580	0.001		**5.402**	19.162	16.345
	40	3.338	>1000.0		1.341	183.921		6.794	0.002		**7.695**	23.592	16.882
	50	3.857	>1000.0		2.407	225.704		8.525	0.002		**9.687**	24.898	17.007
	60	5.804	823.022		3.226	362.775		11.174	0.002		**12.123**	36.212	11.447
150	30	0.055	>1000.0		0.326	106.602		4.141	0.002		**4.990**	39.013	21.052
	40	0.028	>1000.0		0.745	156.994		5.872	0.002		**6.830**	49.541	18.100
	50	0.011	>1000.0		1.009	182.630		7.570	0.002		**8.618**	59.876	17.433
	60	0	>1000.0		1.521	646.268		9.371	0.005		**10.445**	58.715	15.019
	70	0	>1000.0		2.464	1028.650		11.446	0.005		**12.573**	60.901	10.934
	80	0	>1000.0		3.036	549.900		13.611	0.003		**14.744**	64.143	11.666
	90	0	>1000.0		4.193	906.030		15.589	0.005		**16.600**	59.969	13.109
200	40	0	>1000.0		0.370	81.750		5.486	0.005		**6.486**	81.318	15.772
	50	0	>1000.0		0.483	186.900		6.848	0.006		**7.760**	91.498	7.205
	60	0	>1000.0		0.917	313.850		8.710	0.008		**9.635**	82.300	12.738
	70	0	>1000.0		1.680	3112.950		10.395	0.008		**11.252**	81.530	17.200
	80	0	>1000.0		1.795	1629.400		12.529	0.003		**13.415**	88.926	10.166
	90	0	>1000.0		2.045	1364.400		14.046	0.009		**14.693**	77.539	23.186
	100	0	>1000.0		3.098	3024.830		15.993	0.009		**16.941**	82.575	14.761
250	50	0	>1000.0		0.329	557.676		6.783	0.008		**7.536**	103.797	23.062
	60	0	>1000.0		0.945	1400.788		8.285	0.010		**9.021**	99.990	30.057
	70	0	>1000.0		1.326	2380.366		9.699	0.013		**10.499**	97.342	29.580
	80	0	>1000.0		1.445	647.763		11.571	0.013		**12.096**	52.149	49.798
	90	0	>1000.0		1.591	1242.663		12.978	0.016		**13.589**	70.928	50.748
	100	0	>1000.0		2.443	2210.880		14.800	0.016		**15.392**	41.700	53.728
	120	0	>1000.0		2.781	2249.350		18.418	0.018		**18.895**	41.508	52.769
	140	0	>1000.0		4.713	6624.596		22.514	0.020		**23.123**	25.103	41.312
**Avg**					1.757	984.143		9.515	0.006		**10.321**	51.483	20.125

**Table 3:** Numerical results for the instances of Set 2 (random geometric graphs).

*n*	*r*	CPLEX			GH-MWDDS${}^{+}$			PBIG		
		Value	Gap (%)		Value	Time (s)		Value	Time (s)	$\sigma_{\mathrm{Time}}$
100	0.2	**1.116**	0.000		1.064	0.000		**1.116**	0.051	0.088
	0.225	**1.451**	0.000		1.354	0.000		**1.451**	1.933	4.674
	0.25	**1.848**	0.000		1.763	0.000		**1.848**	0.586	1.657
	0.275	**2.610**	0.204		2.462	0.001		**2.610**	1.040	3.835
	0.3	**3.047**	7.640		2.931	0.001		**3.047**	2.826	9.378
500	0.1	0.021	>1000.0		1.004	0.006		**1.037**	5.428	17.623
	0.125	0.000	>1000.0		1.922	0.011		**2.012**	2.490	5.020
	0.15	0.000	>1000.0		3.318	0.016		**3.606**	28.489	41.489
	0.175	0.000	>1000.0		4.517	0.021		**4.929**	43.215	67.606
	0.2	0.000	>1000.0		6.634	0.029		**7.141**	82.951	81.343
1000	0.05	0.000	>1000.0		0.262	0.014		**0.266**	0.373	1.093
	0.075	0.000	>1000.0		1.243	0.030		**1.369**	12.425	20.908
	0.1	0.000	>1000.0		2.721	0.056		**3.083**	83.973	106.192
	0.125	0.000	>1000.0		4.791	0.092		**5.199**	196.269	187.676
	0.15	0.000	>1000.0		7.680	0.141		**8.295**	363.599	136.273
**Avg**					2.911	0.028		**3.134**	55.043	45.657

**Table 4:** Detailed textual description of the solutions displayed in Figure 2 and Figure 3.

Solution of GH-MWDDS${}^{+}$ for the 1st RGG Graph with 100 Nodes and r=0.2; see Figure 2a.				
ID disjoint set	**Color (Figure 2a)**	**#Nodes**	**Lifetime**	**Node IDs**
1	blue	13	0.681	7, 27, 36, 43, 60, 62, 71, 72, 78, 82, 88, 89, 91
2	red	14	0.564	2, 5, 6, 8, 10, 12, 20, 37, 76, 79, 80, 87, 95, 97
3	light green	15	0.185	15, 28, 32, 40, 44, 46, 50, 54, 56, 67, 69, 75, 81, 86, 98
unused nodes	purple	58		0, 1, 3, 4, 9, 11, 13, 14, 16, 17, 18, 19, 21, 22, 23, 24, 25, 26, 29, 30, 31, 33, 34, 35, 38, 39, 41, 42, 45, 47, 48, 49, 51, 52, 53, 55, 57, 58, 59, 61, 63, 64, 65, 66, 68, 70, 73, 74, 77, 83, 84, 85, 90, 92, 93, 94, 96, 99
Best solution of PBIG for the first RGG graph with 100 nodes and r=0.2; see Figure 2b.				
ID disjoint set	Color (Figure 2b)	#Nodes	Lifetime	Node IDs
1	blue	14	0.754	12, 27, 36, 37, 43, 60, 62, 71, 78, 80, 82, 88, 89, 91
2	dark green	13	0.564	2, 5, 6, 7, 8, 10, 20, 72, 79, 87, 95, 97, 98
3	light green	15	0.185	15, 28, 32, 40, 44, 46, 50, 54, 56, 63, 67, 75, 81, 83, 86
4	red	14	0.160	16, 24, 31, 42, 51, 53, 57, 59, 68, 69, 76, 85, 90, 96
unused nodes	purple	44		0, 1, 3, 4, 9, 11, 13, 14, 17, 18, 19, 21, 22, 23, 25, 26, 29, 30, 33, 34, 35, 38, 39, 41, 45, 47, 48, 49, 52, 55, 58, 61, 64, 65, 66, 70, 73, 74, 77, 84, 92, 93, 94, 99
Solution of GH-MWDDS${}^{+}$ for the first RGG graph with 100 nodes and r=0.3; see Figure 3a.				
ID disjoint set	Color (Figure 3a)	#Nodes	Lifetime	Node IDs
1	dark green	6	0.715	26, 27, 58, 64, 73, 95
2	light green	9	0.657	1, 6, 20, 24, 40, 51, 83, 88, 94
3	red	7	0.588	12, 25, 72, 76, 89, 91, 98
4	blue	9	0.532	2, 15, 38, 39, 61, 75, 79, 93, 99
5	brown	8	0.337	8, 18, 21, 36, 44, 46, 90, 92
6	orange	8	0.079	14, 17, 43, 54, 55, 81, 84, 85
unused nodes	purple	53		0, 3, 4, 5, 7, 9, 10, 11, 13, 16, 19, 22, 23, 28, 29, 30, 31, 32, 33, 34, 35, 37, 41, 42, 45, 47, 48, 49, 50, 52, 53, 56, 57, 59, 60, 62, 63, 65, 66, 67, 68, 69, 70, 71, 74, 77, 78, 80, 82, 86, 87, 96, 97
Best solution of PBIG for the first RGG graph with 100 nodes and r=0.3; see Figure 3b.				
ID disjoint set	Color (Figure 3b)	#Nodes	Lifetime	Node IDs
1	blue	8	0.834	6, 20, 22, 26, 27, 51, 73, 95
2	red	7	0.736	12, 25, 58, 72, 76, 88, 94
3	orange	8	0.532	1, 2, 15, 24, 36, 38, 93, 98
4	dark green	6	0.588	44, 61, 64, 74, 83, 89
5	brown	8	0.337	8, 18, 21, 39, 46, 90, 91, 92
6	light green	9	0.079	14, 17, 43, 75, 79, 81, 84, 85, 99
unused nodes	purple	54		0, 3, 4, 5, 7, 9, 10, 11, 13, 16, 19, 23, 28, 29, 30, 31, 32, 33, 34, 35, 37, 40, 41, 42, 45, 47, 48, 49, 50, 52, 53, 54, 55, 56, 57, 59, 60, 62, 63, 65, 66, 67, 68, 69, 70, 71, 77, 78, 80, 82, 86, 87, 96, 97

## Data Availability

Both benchmark sets used in this work can be obtained at https://www.iiia.csic.es/~christian.blum/research.html#Instances (accessed on accessed on 16 January 2022).

## Abbreviations

The following abbreviations are used in this manuscript:

PBIG	Population-Based Iterated Greedy
MDDS	Maximum Disjoint Dominating Sets
MWDDS	Maximum Weighted Disjoint Dominating Sets
